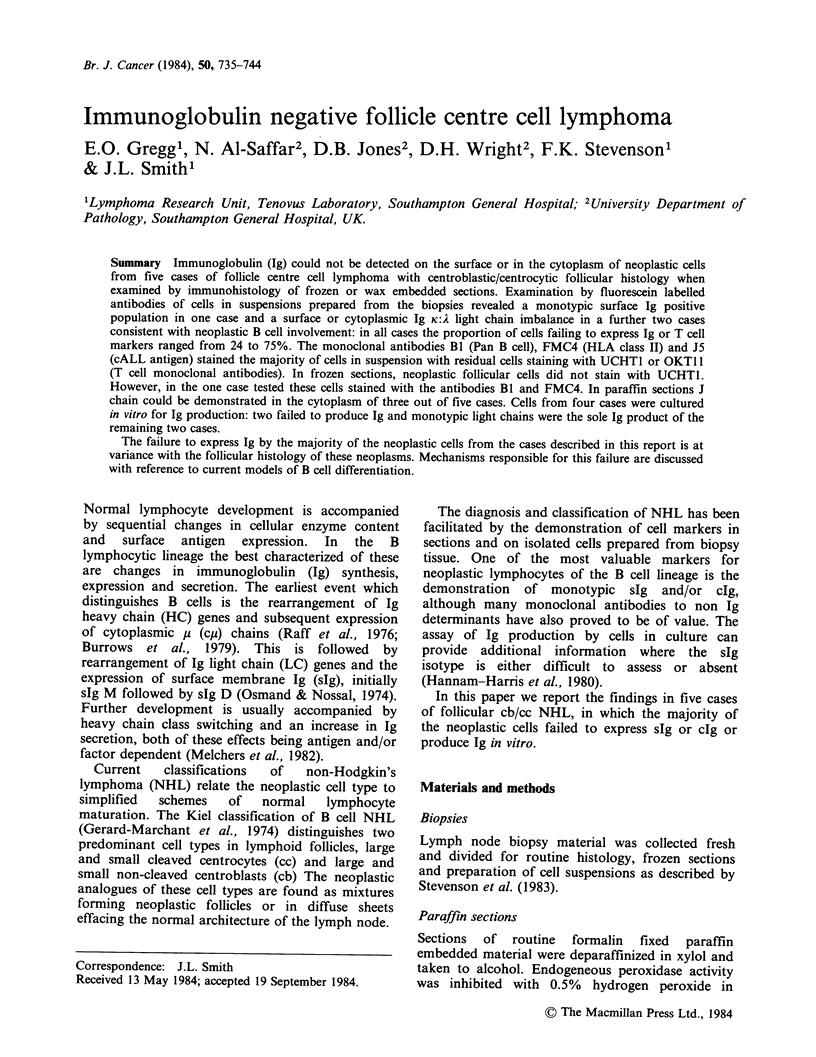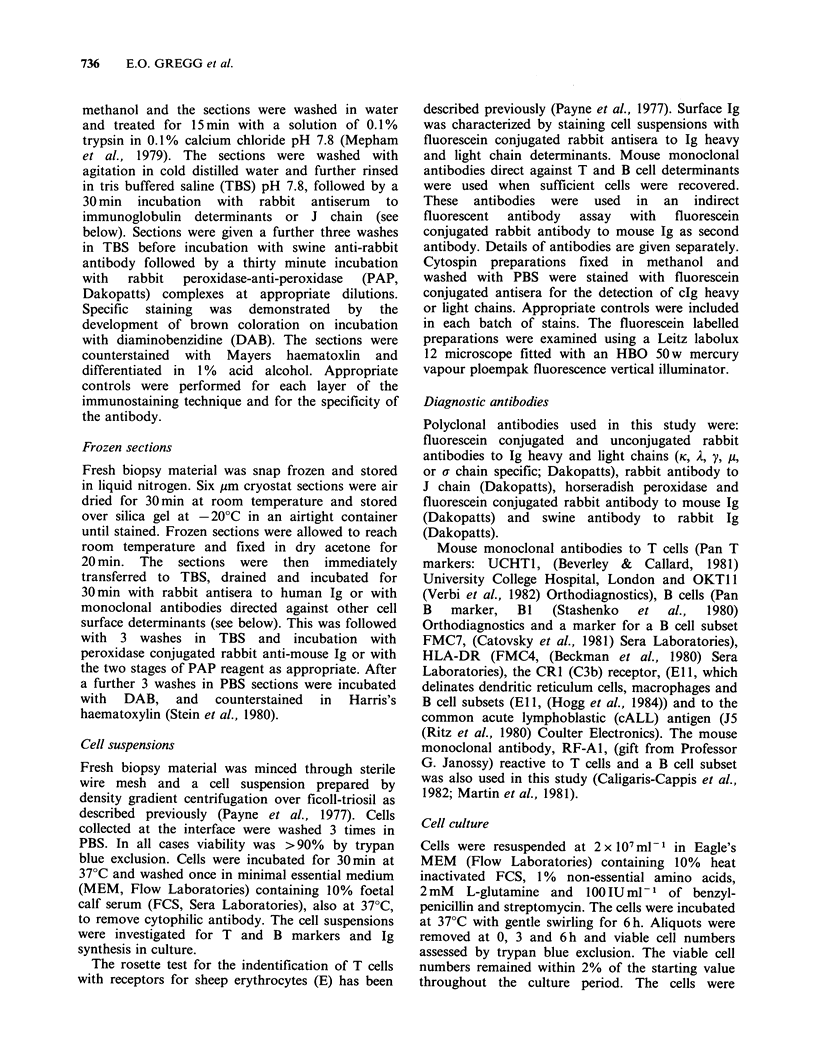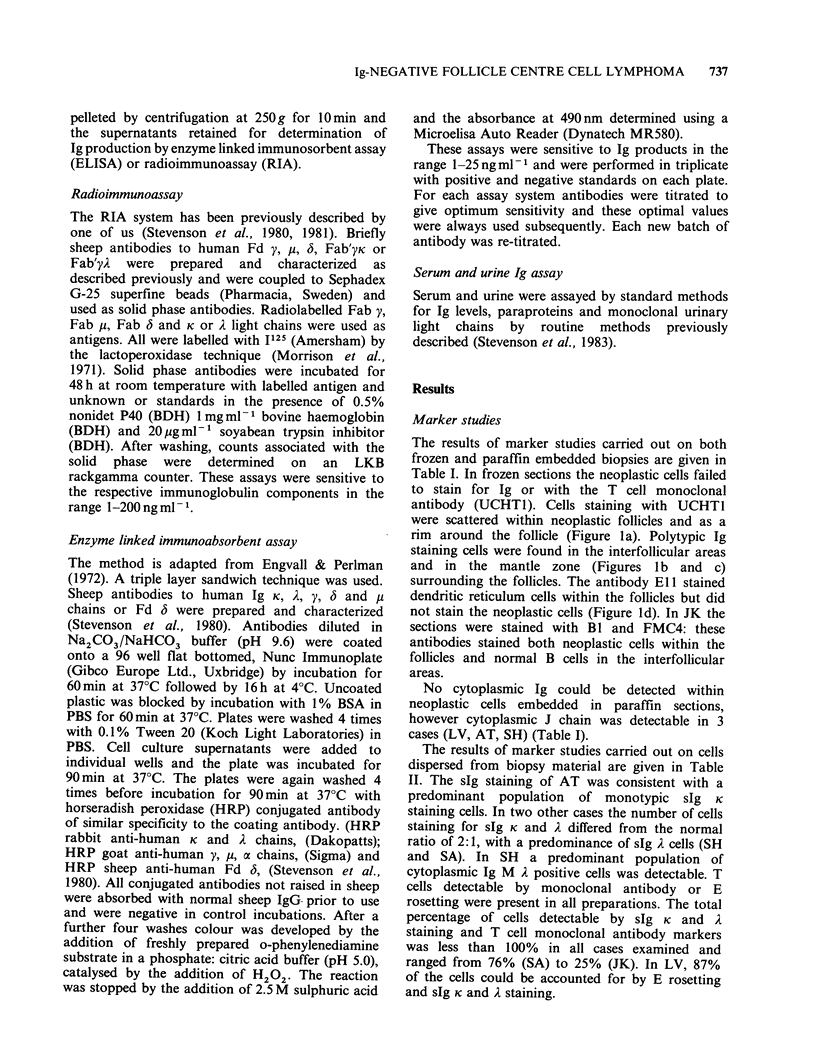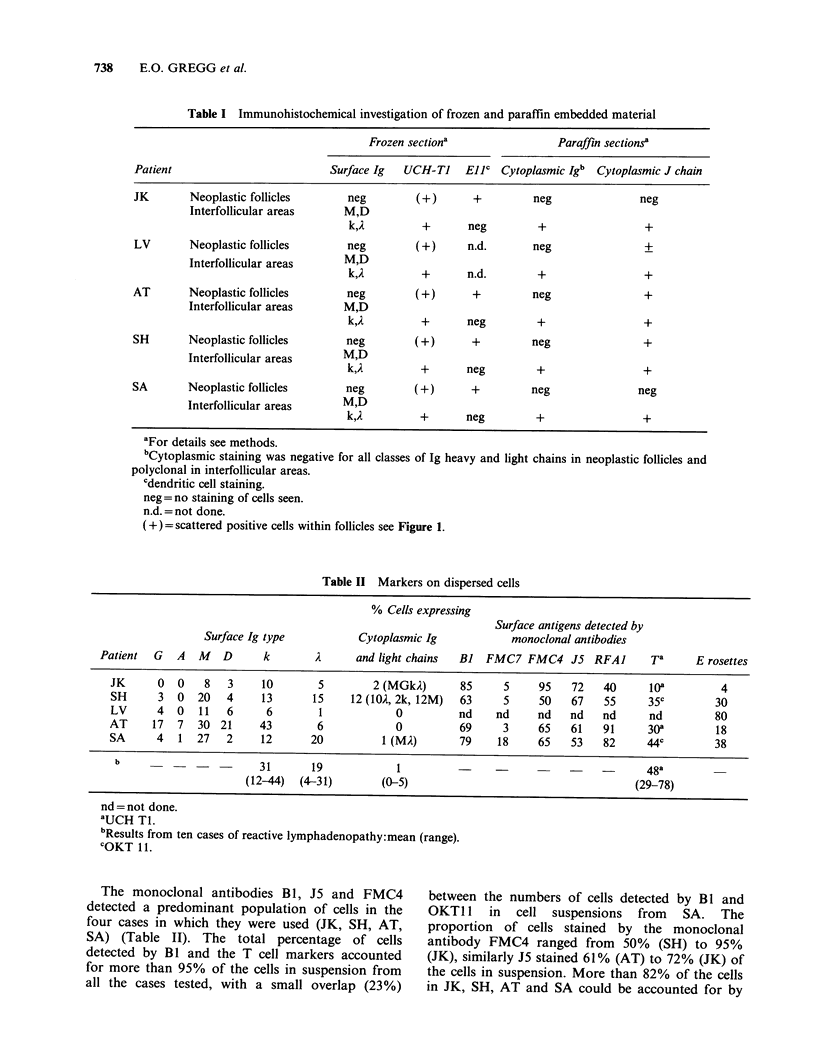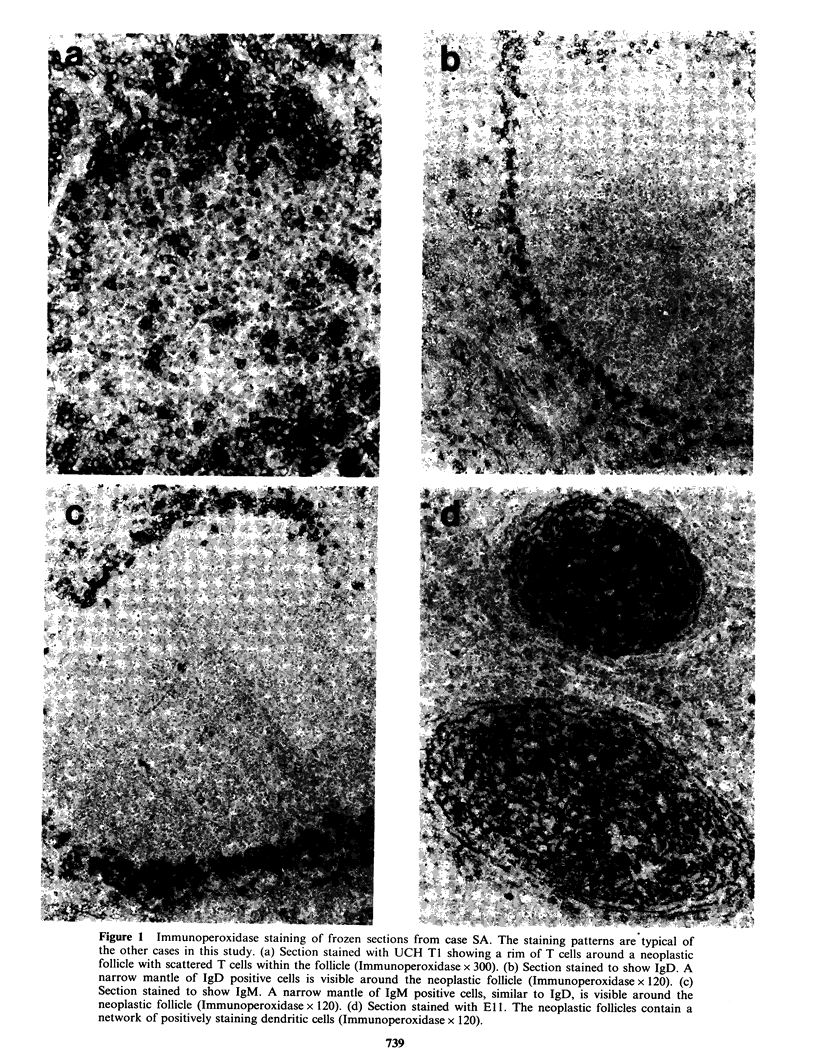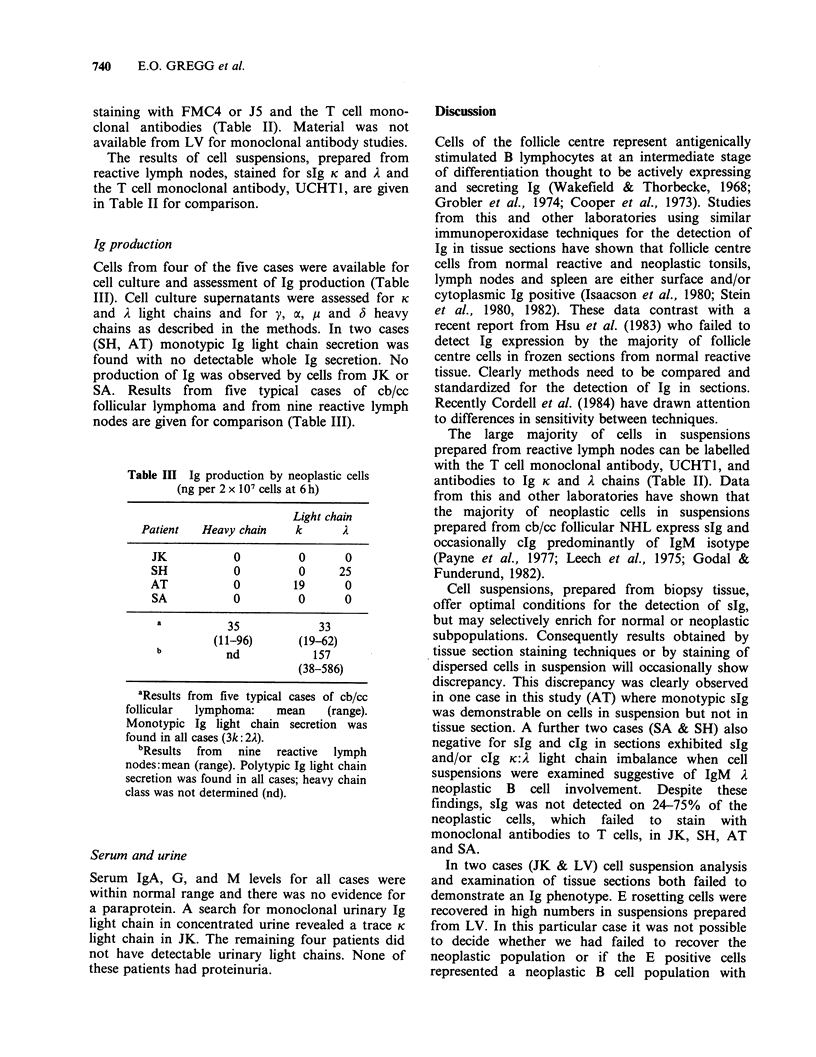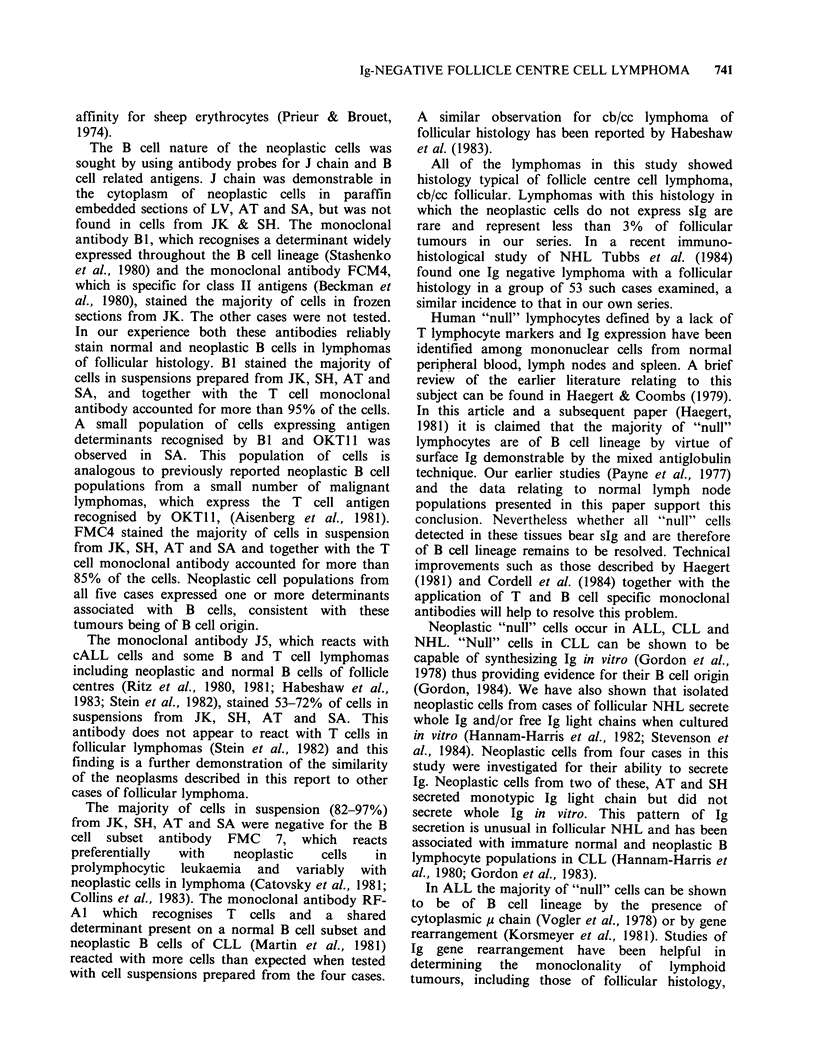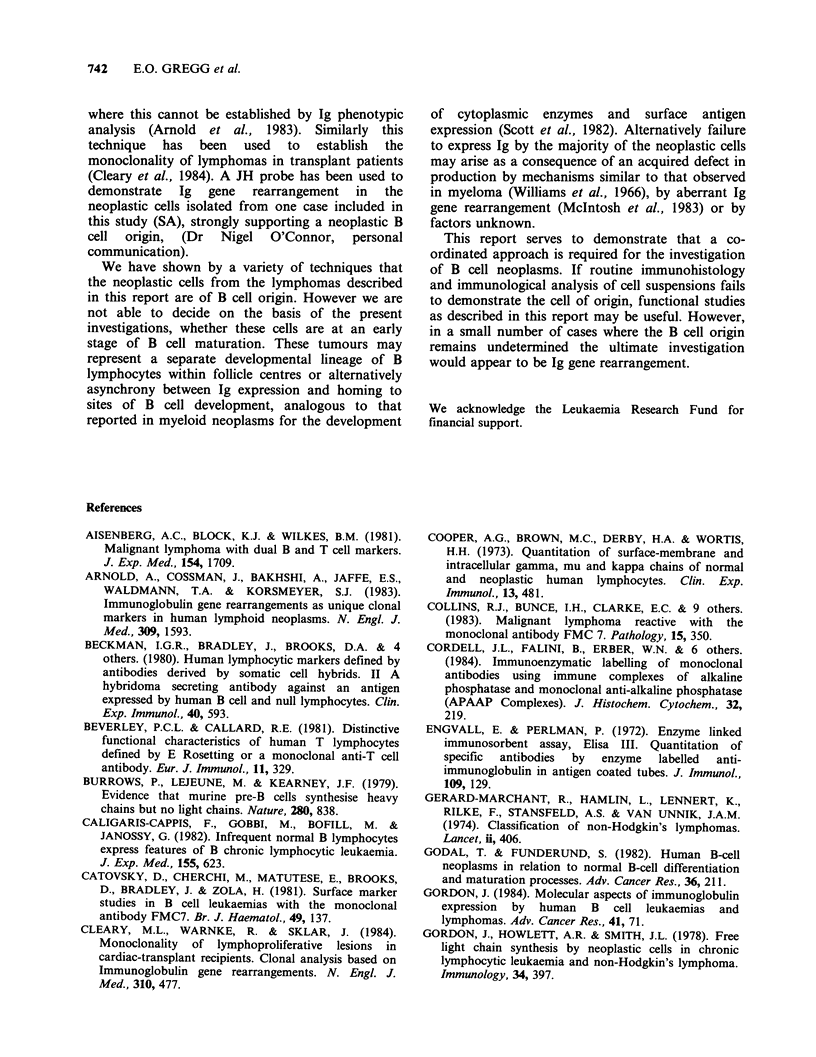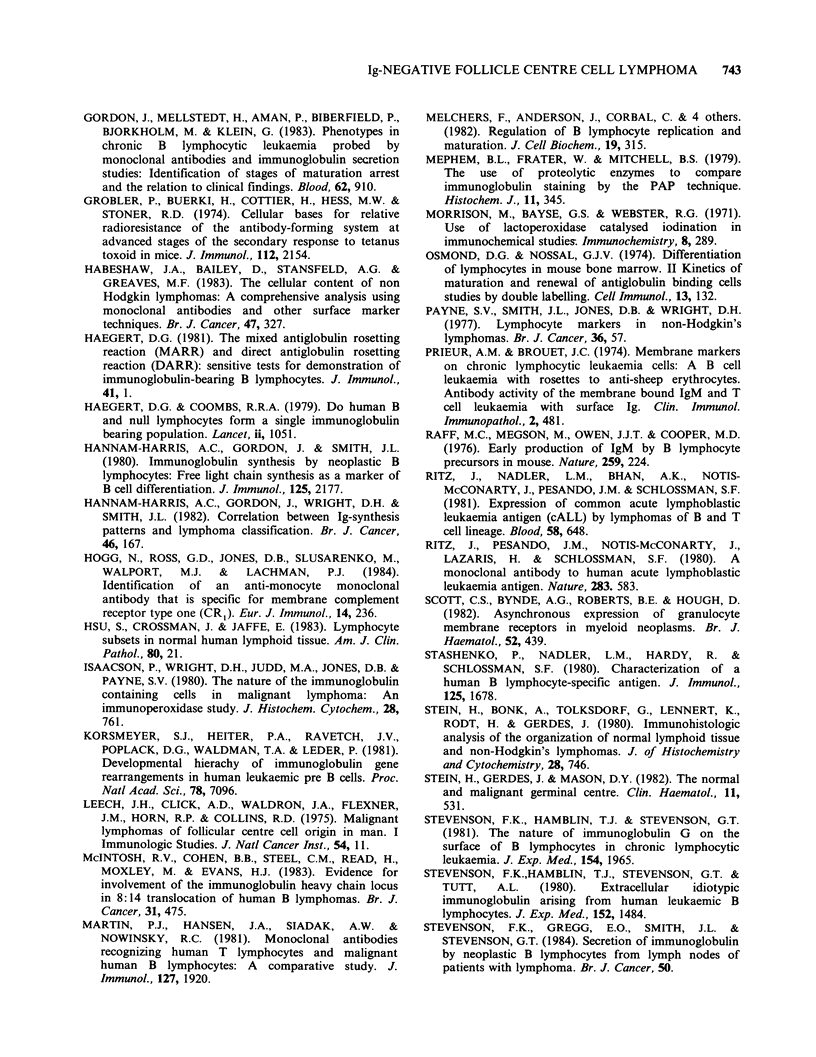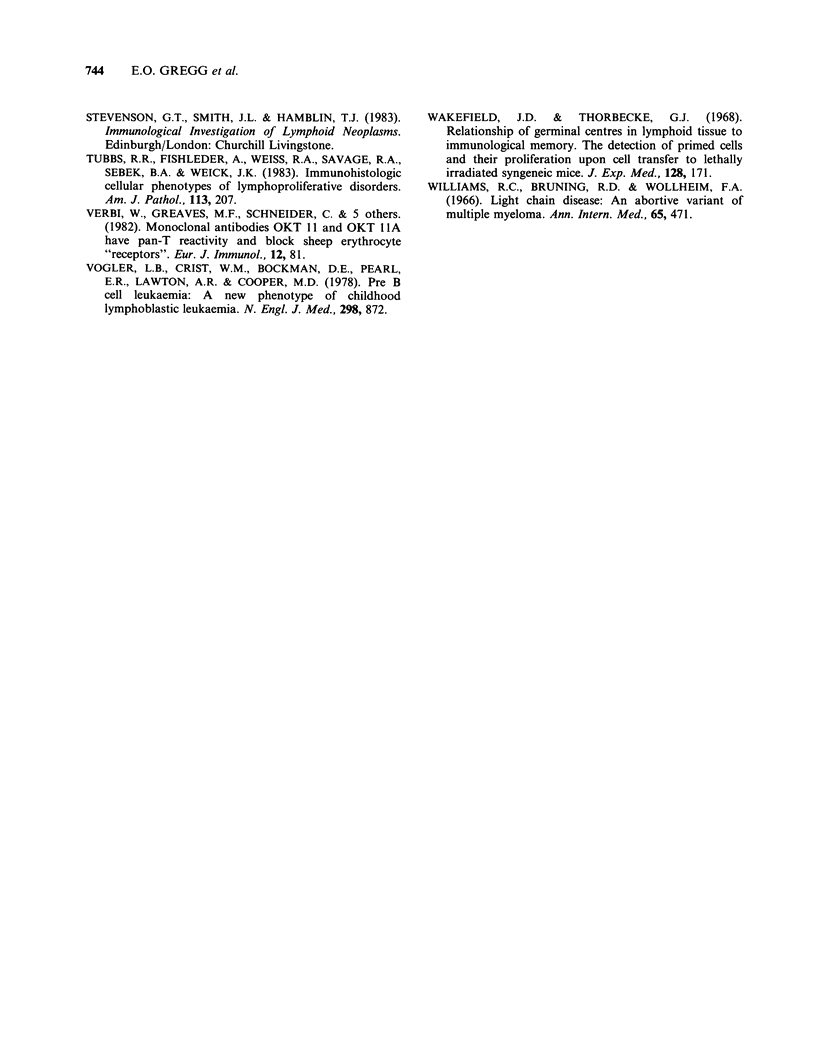# Immunoglobulin negative follicle centre cell lymphoma.

**DOI:** 10.1038/bjc.1984.250

**Published:** 1984-12

**Authors:** E. O. Gregg, N. Al-Saffar, D. B. Jones, D. H. Wright, F. K. Stevenson, J. L. Smith

## Abstract

**Images:**


*Br. J. Cancer* (1984), **50**, 735–744

### Immunoglobulin negative follicle centre cell lymphoma

E.O. Gregg[1], N. Al-Saffar[2], D.B. Jones[2], D.H. Wright[2], F.K. Stevenson[1] & J.L. Smith[1]

[1]*Lymphoma Research Unit, Tenovus Laboratory, Southampton General Hospital;* [2]*University Department of Pathology, Southampton General Hospital, UK.*

**Summary** Immunoglobulin (Ig) could not be detected on the surface or in the cytoplasm of neoplastic cells from five cases of follicle centre cell lymphoma with centroblastic/centrocytic follicular histology when examined by immunohistology of frozen or wax embedded sections. Examination by fluorescein labelled antibodies of cells in suspensions prepared from the biopsies revealed a monotypic surface Ig positive population in one case and a surface or cytoplasmic Ig $\kappa:\lambda$ light chain imbalance in a further two cases consistent with neoplastic B cell involvement: in all cases the proportion of cells failing to express Ig or T cell markers ranged from 24 to 75%. The monoclonal antibodies B1 (Pan B cell), FMC4 (HLA class II) and J5 (cALL antigen) stained the majority of cells in suspension with residual cells staining with UCHT1 or OKT11 (T cell monoclonal antibodies). In frozen sections, neoplastic follicular cells did not stain with UCHT1. However, in the one case tested these cells stained with the antibodies B1 and FMC4. In paraffin sections J chain could be demonstrated in the cytoplasm of three out of five cases. Cells from four cases were cultured *in vitro* for Ig production: two failed to produce Ig and monotypic light chains were the sole Ig product of the remaining two cases.

The failure to express Ig by the majority of the neoplastic cells from the cases described in this report is at variance with the follicular histology of these neoplasms. Mechanisms responsible for this failure are discussed with reference to current models of B cell differentiation.

Normal lymphocyte development is accompanied by sequential changes in cellular enzyme content and surface antigen expression. In the B lymphocytic lineage the best characterized of these are changes in immunoglobulin (Ig) synthesis, expression and secretion. The earliest event which distinguishes B cells is the rearrangement of Ig heavy chain (HC) genes and subsequent expression of cytoplasmic $\mu$ (c$\mu$) chains (Raff *et al.*, 1976; Burrows *et al.*, 1979). This is followed by rearrangement of Ig light chain (LC) genes and the expression of surface membrane Ig (sIg), initially sIg M followed by sIg D (Osmand & Nossal, 1974). Further development is usually accompanied by heavy chain class switching and an increase in Ig secretion, both of these effects being antigen and/or factor dependent (Melchers *et al.*, 1982).

Current classifications of non-Hodgkin's lymphoma (NHL) relate the neoplastic cell type to simplified schemes of normal lymphocyte maturation. The Kiel classification of B cell NHL (Gerard-Marchant *et al.*, 1974) distinguishes two predominant cell types in lymphoid follicles, large and small cleaved centrocytes (cc) and large and small non-cleaved centroblasts (cb) The neoplastic analogues of these cell types are found as mixtures forming neoplastic follicles or in diffuse sheets effacing the normal architecture of the lymph node.

Correspondence: J.L. Smith
Received 13 May 1984; accepted 19 September 1984.

The diagnosis and classification of NHL has been facilitated by the demonstration of cell markers in sections and on isolated cells prepared from biopsy tissue. One of the most valuable markers for neoplastic lymphocytes of the B cell lineage is the demonstration of monotypic sIg and/or cIg, although many monoclonal antibodies to non Ig determinants have also proved to be of value. The assay of Ig production by cells in culture can provide additional information where the sIg isotype is either difficult to assess or absent (Hannam-Harris *et al.*, 1980).

In this paper we report the findings in five cases of follicular cb/cc NHL, in which the majority of the neoplastic cells failed to express sIg or cIg or produce Ig *in vitro*.

### Materials and methods

### *Biopsies*

Lymph node biopsy material was collected fresh and divided for routine histology, frozen sections and preparation of cell suspensions as described by Stevenson *et al.* (1983).

### *Paraffin sections*

Sections of routine formalin fixed paraffin embedded material were deparaffinized in xylol and taken to alcohol. Endogeneous peroxidase activity was inhibited with 0.5% hydrogen peroxide in

© The Macmillan Press Ltd., 1984

methanol and the sections were washed in water and treated for 15 min with a solution of 0.1% trypsin in 0.1% calcium chloride pH 7.8 (Mepham *et al.*, 1979). The sections were washed with agitation in cold distilled water and further rinsed in tris buffered saline (TBS) pH 7.8, followed by a 30 min incubation with rabbit antiserum to immunoglobulin determinants or J chain (see below). Sections were given a further three washes in TBS before incubation with swine anti-rabbit antibody followed by a thirty minute incubation with rabbit peroxidase-anti-peroxidase (PAP, Dakopatts) complexes at appropriate dilutions. Specific staining was demonstrated by the development of brown coloration on incubation with diaminobenzidine (DAB). The sections were counterstained with Mayers haematoxlin and differentiated in 1% acid alcohol. Appropriate controls were performed for each layer of the immunostaining technique and for the specificity of the antibody.

*Frozen sections*

Fresh biopsy material was snap frozen and stored in liquid nitrogen. Six $\mu$m cryostat sections were air dried for 30 min at room temperature and stored over silica gel at $-20°C$ in an airtight container until stained. Frozen sections were allowed to reach room temperature and fixed in dry acetone for 20 min. The sections were then immediately transferred to TBS, drained and incubated for 30 min with rabbit antisera to human Ig or with monoclonal antibodies directed against other cell surface determinants (see below). This was followed with 3 washes in TBS and incubation with peroxidase conjugated rabbit anti-mouse Ig or with the two stages of PAP reagent as appropriate. After a further 3 washes in PBS sections were incubated with DAB, and counterstained in Harris's haematoxylin (Stein *et al.*, 1980).

*Cell suspensions*

Fresh biopsy material was minced through sterile wire mesh and a cell suspension prepared by density gradient centrifugation over ficoll-triosil as described previously (Payne *et al.*, 1977). Cells collected at the interface were washed 3 times in PBS. In all cases viability was >90% by trypan blue exclusion. Cells were incubated for 30 min at 37°C and washed once in minimal essential medium (MEM, Flow Laboratories) containing 10% foetal calf serum (FCS, Sera Laboratories), also at 37°C, to remove cytophilic antibody. The cell suspensions were investigated for T and B markers and Ig synthesis in culture.

The rosette test for the indentification of T cells with receptors for sheep erythrocytes (E) has been described previously (Payne *et al.*, 1977). Surface Ig was characterized by staining cell suspensions with fluorescein conjugated rabbit antisera to Ig heavy and light chain determinants. Mouse monoclonal antibodies direct against T and B cell determinants were used when sufficient cells were recovered. These antibodies were used in an indirect fluorescent antibody assay with fluorescein conjugated rabbit antibody to mouse Ig as second antibody. Details of antibodies are given separately. Cytospin preparations fixed in methanol and washed with PBS were stained with fluorescein conjugated antisera for the detection of cIg heavy or light chains. Appropriate controls were included in each batch of stains. The fluorescein labelled preparations were examined using a Leitz labolux 12 microscope fitted with an HBO 50 w mercury vapour ploempak fluorescence vertical illuminator.

*Diagnostic antibodies*

Polyclonal antibodies used in this study were: fluorescein conjugated and unconjugated rabbit antibodies to Ig heavy and light chains ($\kappa$, $\lambda$, $\gamma$, $\mu$, or $\sigma$ chain specific; Dakopatts), rabbit antibody to J chain (Dakopatts), horseradish peroxidase and fluorescein conjugated rabbit antibody to mouse Ig (Dakopatts) and swine antibody to rabbit Ig (Dakopatts).

Mouse monoclonal antibodies to T cells (Pan T markers: UCHT1, (Beverley & Callard, 1981) University College Hospital, London and OKT11 (Verbi *et al.*, 1982) Orthodiagnostics), B cells (Pan B marker, B1 (Stashenko *et al.*, 1980) Orthodiagnostics and a marker for a B cell subset FMC7, (Catovsky *et al.*, 1981) Sera Laboratories), HLA-DR (FMC4, (Beckman *et al.*, 1980) Sera Laboratories), the CR1 (C3b) receptor, (E11, which delinates dendritic reticulum cells, macrophages and B cell subsets (E11, (Hogg *et al.*, 1984)) and to the common acute lymphoblastic (cALL) antigen (J5 (Ritz *et al.*, 1980) Coulter Electronics). The mouse monoclonal antibody, RF-A1, (gift from Professor G. Janossy) reactive to T cells and a B cell subset was also used in this study (Caligaris-Cappis *et al.*, 1982; Martin *et al.*, 1981).

*Cell culture*

Cells were resuspended at $2 \times 10^7 ml^{-1}$ in Eagle's MEM (Flow Laboratories) containing 10% heat inactivated FCS, 1% non-essential amino acids, 2 mM L-glutamine and 100 IU $ml^{-1}$ of benzyl-penicillin and streptomycin. The cells were incubated at 37°C with gentle swirling for 6 h. Aliquots were removed at 0, 3 and 6 h and viable cell numbers assessed by trypan blue exclusion. The viable cell numbers remained within 2% of the starting value throughout the culture period. The cells were pelleted by centrifugation at 250 *g* for 10 min and the supernatants retained for determination of Ig production by enzyme linked immunosorbent assay (ELISA) or radioimmunoassay (RIA).

*Radioimmunoassay*

The RIA system has been previously described by one of us (Stevenson *et al.*, 1980, 1981). Briefly sheep antibodies to human Fd $\gamma$, $\mu$, $\delta$, Fab'$\gamma\kappa$ or Fab'$\gamma\lambda$ were prepared and characterized as described previously and were coupled to Sephadex G-25 superfine beads (Pharmacia, Sweden) and used as solid phase antibodies. Radiolabelled Fab $\gamma$, Fab $\mu$, Fab $\delta$ and $\kappa$ or $\lambda$ light chains were used as antigens. All were labelled with $I^{125}$ (Amersham) by the lactoperoxidase technique (Morrison *et al.*, 1971). Solid phase antibodies were incubated for 48 h at room temperature with labelled antigen and unknown or standards in the presence of 0.5% nonidet P40 (BDH) 1 mg ml$^{-1}$ bovine haemoglobin (BDH) and 20 $\mu$g ml$^{-1}$ soyabean trypsin inhibitor (BDH). After washing, counts associated with the solid phase were determined on an LKB rackgamma counter. These assays were sensitive to the respective immunoglobulin components in the range 1–200 ng ml$^{-1}$.

*Enzyme linked immunoabsorbent assay*

The method is adapted from Engvall & Perlman (1972). A triple layer sandwich technique was used. Sheep antibodies to human Ig $\kappa$, $\lambda$, $\gamma$, $\delta$ and $\mu$ chains or Fd $\delta$ were prepared and characterized (Stevenson *et al.*, 1980). Antibodies diluted in $Na_2CO_3/NaHCO_3$ buffer (pH 9.6) were coated onto a 96 well flat bottomed, Nunc Immunoplate (Gibco Europe Ltd., Uxbridge) by incubation for 60 min at 37°C followed by 16 h at 4°C. Uncoated plastic was blocked by incubation with 1% BSA in PBS for 60 min at 37°C. Plates were washed 4 times with 0.1% Tween 20 (Koch Light Laboratories) in PBS. Cell culture supernatants were added to individual wells and the plate was incubated for 90 min at 37°C. The plates were again washed 4 times before incubation for 90 min at 37°C with horseradish peroxidase (HRP) conjugated antibody of similar specificity to the coating antibody. (HRP rabbit anti-human $\kappa$ and $\lambda$ chains, (Dakopatts); HRP goat anti-human $\gamma$, $\mu$, $\alpha$ chains, (Sigma) and HRP sheep anti-human Fd $\delta$, (Stevenson *et al.*, 1980). All conjugated antibodies not raised in sheep were absorbed with normal sheep IgG prior to use and were negative in control incubations. After a further four washes colour was developed by the addition of freshly prepared o-phenylenediamine substrate in a phosphate: citric acid buffer (pH 5.0), catalysed by the addition of $H_2O_2$. The reaction was stopped by the addition of 2.5 M sulphuric acid and the absorbance at 490 nm determined using a Microelisa Auto Reader (Dynatech MR580).

These assays were sensitive to Ig products in the range 1–25 ng ml$^{-1}$ and were performed in triplicate with positive and negative standards on each plate. For each assay system antibodies were titrated to give optimum sensitivity and these optimal values were always used subsequently. Each new batch of antibody was re-titrated.

*Serum and urine Ig assay*

Serum and urine were assayed by standard methods for Ig levels, paraproteins and monoclonal urinary light chains by routine methods previously described (Stevenson *et al.*, 1983).

### Results

*Marker studies*

The results of marker studies carried out on both frozen and paraffin embedded biopsies are given in Table I. In frozen sections the neoplastic cells failed to stain for Ig or with the T cell monoclonal antibody (UCHT1). Cells staining with UCHT1 were scattered within neoplastic follicles and as a rim around the follicle (Figure 1a). Polytypic Ig staining cells were found in the interfollicular areas and in the mantle zone (Figures 1b and c) surrounding the follicles. The antibody E11 stained dendritic reticulum cells within the follicles but did not stain the neoplastic cells (Figure 1d). In JK the sections were stained with B1 and FMC4: these antibodies stained both neoplastic cells within the follicles and normal B cells in the interfollicular areas.

No cytoplasmic Ig could be detected within neoplastic cells embedded in paraffin sections, however cytoplasmic J chain was detectable in 3 cases (LV, AT, SH) (Table I).

The results of marker studies carried out on cells dispersed from biopsy material are given in Table II. The sIg staining of AT was consistent with a predominant population of monotypic sIg $\kappa$ staining cells. In two other cases the number of cells staining for sIg $\kappa$ and $\lambda$ differed from the normal ratio of 2:1, with a predominance of sIg $\lambda$ cells (SH and SA). In SH a predominant population of cytoplasmic Ig M $\lambda$ positive cells was detectable. T cells detectable by monoclonal antibody or E rosetting were present in all preparations. The total percentage of cells detectable by sIg $\kappa$ and $\lambda$ staining and T cell monoclonal antibody markers was less than 100% in all cases examined and ranged from 76% (SA) to 25% (JK). In LV, 87% of the cells could be accounted for by E rosetting and sIg $\kappa$ and $\lambda$ staining.

**Table I** Immunohistochemical investigation of frozen and paraffin embedded material

| Patient | | Frozen section[a] | | | Paraffin sections[a] | |
|---|---|---|---|---|---|---|
| | | *Surface Ig* | *UCH-T1* | *E11*[c] | *Cytoplasmic Ig*[b] | *Cytoplasmic J chain* |
| JK | Neoplastic follicles | neg | (+) | + | neg | neg |
| | Interfollicular areas | M,D k,λ | + | neg | + | + |
| LV | Neoplastic follicles | neg | (+) | n.d. | neg | ± |
| | Interfollicular areas | M,D k,λ | + | n.d. | + | + |
| AT | Neoplastic follicles | neg | (+) | + | neg | + |
| | Interfollicular areas | M,D k,λ | + | neg | + | + |
| SH | Neoplastic follicles | neg | (+) | + | neg | + |
| | Interfollicular areas | M,D k,λ | + | neg | + | + |
| SA | Neoplastic follicles | neg | (+) | + | neg | neg |
| | Interfollicular areas | M,D k,λ | + | neg | + | + |

[a]For details see methods.
[b]Cytoplasmic staining was negative for all classes of Ig heavy and light chains in neoplastic follicles and polyclonal in interfollicular areas.
[c]dendritic cell staining.
neg = no staining of cells seen.
n.d. = not done.
(+) = scattered positive cells within follicles see **Figure 1**.

**Table II** Markers on dispersed cells

| | % Cells expressing | | | | | | | | | | | | | | |
|---|---|---|---|---|---|---|---|---|---|---|---|---|---|---|---|
| | *Surface Ig type* | | | | | | *Cytoplasmic Ig* | | *Surface antigens detected by monoclonal antibodies* | | | | | | |
| *Patient* | *G* | *A* | *M* | *D* | *k* | *λ* | *and light chains* | *B1* | *FMC7* | *FMC4* | *J5* | *RFA1* | *T*[a] | *E rosettes* |
| JK | 0 | 0 | 8 | 3 | 10 | 5 | 2 (MGkλ) | 85 | 5 | 95 | 72 | 40 | 10[a] | 4 |
| SH | 3 | 0 | 20 | 4 | 13 | 15 | 12 (10λ, 2k, 12M) | 63 | 5 | 50 | 67 | 55 | 35[c] | 30 |
| LV | 4 | 0 | 11 | 6 | 6 | 1 | 0 | nd | nd | nd | nd | nd | nd | 80 |
| AT | 17 | 7 | 30 | 21 | 43 | 6 | 0 | 69 | 3 | 65 | 61 | 91 | 30[a] | 18 |
| SA | 4 | 1 | 27 | 2 | 12 | 20 | 1 (Mλ) | 79 | 18 | 65 | 53 | 82 | 44[c] | 38 |
| [b] | — | — | — | — | 31 (12–44) | 19 (4–31) | 1 (0–5) | — | — | — | — | — | 48[a] (29–78) | — |

nd = not done.
[a]UCH T1.
[b]Results from ten cases of reactive lymphadenopathy:mean (range).
[c]OKT 11.

The monoclonal antibodies B1, J5 and FMC4 detected a predominant population of cells in the four cases in which they were used (JK, SH, AT, SA) (Table II). The total percentage of cells detected by B1 and the T cell markers accounted for more than 95% of the cells in suspension from all the cases tested, with a small overlap (23%) between the numbers of cells detected by B1 and OKT11 in cell suspensions from SA. The proportion of cells stained by the monoclonal antibody FMC4 ranged from 50% (SH) to 95% (JK), similarly J5 stained 61% (AT) to 72% (JK) of the cells in suspension. More than 82% of the cells in JK, SH, AT and SA could be accounted for by

**Figure 1** Immunoperoxidase staining of frozen sections from case SA. The staining patterns are typical of the other cases in this study. (a) Section stained with UCH T1 showing a rim of T cells around a neoplastic follicle with scattered T cells within the follicle (Immunoperoxidase × 300). (b) Section stained to show IgD. A narrow mantle of IgD positive cells is visible around the neoplastic follicle (Immunoperoxidase × 120). (c) Section stained to show IgM. A narrow mantle of IgM positive cells, similar to IgD, is visible around the neoplastic follicle (Immunoperoxidase × 120). (d) Section stained with E11. The neoplastic follicles contain a network of positively staining dendritic cells (Immunoperoxidase × 120).

staining with FMC4 or J5 and the T cell monoclonal antibodies (Table II). Material was not available from LV for monoclonal antibody studies.

The results of cell suspensions, prepared from reactive lymph nodes, stained for sIg $\kappa$ and $\lambda$ and the T cell monoclonal antibody, UCHT1, are given in Table II for comparison.

*Ig production*

Cells from four of the five cases were available for cell culture and assessment of Ig production (Table III). Cell culture supernatants were assessed for $\kappa$ and $\lambda$ light chains and for $\gamma$, $\alpha$, $\mu$ and $\delta$ heavy chains as described in the methods. In two cases (SH, AT) monotypic Ig light chain secretion was found with no detectable whole Ig secretion. No production of Ig was observed by cells from JK or SA. Results from five typical cases of cb/cc follicular lymphoma and from nine reactive lymph nodes are given for comparison (Table III).

**Table III** Ig production by neoplastic cells (ng per $2 \times 10^7$ cells at 6 h)

| *Patient* | *Heavy chain* | *Light chain* *k* | *λ* |
|---|---|---|---|
| JK | 0 | 0 | 0 |
| SH | 0 | 0 | 25 |
| AT | 0 | 19 | 0 |
| SA | 0 | 0 | 0 |
| [a] | 35 (11–96) | 33 (19–62) | |
| [b] | nd | 157 (38–586) | |

[a]Results from five typical cases of cb/cc follicular lymphoma: mean (range). Monotypic Ig light chain secretion was found in all cases ($3k:2\lambda$).

[b]Results from nine reactive lymph nodes: mean (range). Polytypic Ig light chain secretion was found in all cases; heavy chain class was not determined (nd).

*Serum and urine*

Serum IgA, G, and M levels for all cases were within normal range and there was no evidence for a paraprotein. A search for monoclonal urinary Ig light chain in concentrated urine revealed a trace $\kappa$ light chain in JK. The remaining four patients did not have detectable urinary light chains. None of these patients had proteinuria.

### Discussion

Cells of the follicle centre represent antigenically stimulated B lymphocytes at an intermediate stage of differentiation thought to be actively expressing and secreting Ig (Wakefield & Thorbecke, 1968; Grobler *et al.,* 1974; Cooper *et al.,* 1973). Studies from this and other laboratories using similar immunoperoxidase techniques for the detection of Ig in tissue sections have shown that follicle centre cells from normal reactive and neoplastic tonsils, lymph nodes and spleen are either surface and/or cytoplasmic Ig positive (Isaacson *et al.,* 1980; Stein *et al.,* 1980, 1982). These data contrast with a recent report from Hsu *et al.* (1983) who failed to detect Ig expression by the majority of follicle centre cells in frozen sections from normal reactive tissue. Clearly methods need to be compared and standardized for the detection of Ig in sections. Recently Cordell *et al.* (1984) have drawn attention to differences in sensitivity between techniques.

The large majority of cells in suspensions prepared from reactive lymph nodes can be labelled with the T cell monoclonal antibody, UCHT1, and antibodies to Ig $\kappa$ and $\lambda$ chains (Table II). Data from this and other laboratories have shown that the majority of neoplastic cells in suspensions prepared from cb/cc follicular NHL express sIg and occasionally cIg predominantly of IgM isotype (Payne *et al.,* 1977; Leech *et al.,* 1975; Godal & Funderund, 1982).

Cell suspensions, prepared from biopsy tissue, offer optimal conditions for the detection of sIg, but may selectively enrich for normal or neoplastic subpopulations. Consequently results obtained by tissue section staining techniques or by staining of dispersed cells in suspension will occasionally show discrepancy. This discrepancy was clearly observed in one case in this study (AT) where monotypic sIg was demonstrable on cells in suspension but not in tissue section. A further two cases (SA & SH) also negative for sIg and cIg in sections exhibited sIg and/or cIg $\kappa:\lambda$ light chain imbalance when cell suspensions were examined suggestive of IgM $\lambda$ neoplastic B cell involvement. Despite these findings, sIg was not detected on 24–75% of the neoplastic cells, which failed to stain with monoclonal antibodies to T cells, in JK, SH, AT and SA.

In two cases (JK & LV) cell suspension analysis and examination of tissue sections both failed to demonstrate an Ig phenotype. E rosetting cells were recovered in high numbers in suspensions prepared from LV. In this particular case it was not possible to decide whether we had failed to recover the neoplastic population or if the E positive cells represented a neoplastic B cell population with affinity for sheep erythrocytes (Prieur & Brouet, 1974).

The B cell nature of the neoplastic cells was sought by using antibody probes for J chain and B cell related antigens. J chain was demonstrable in the cytoplasm of neoplastic cells in paraffin embedded sections of LV, AT and SA, but was not found in cells from JK & SH. The monoclonal antibody B1, which recognises a determinant widely expressed throughout the B cell lineage (Stashenko *et al.*, 1980) and the monoclonal antibody FCM4, which is specific for class II antigens (Beckman *et al.*, 1980), stained the majority of cells in frozen sections from JK. The other cases were not tested. In our experience both these antibodies reliably stain normal and neoplastic B cells in lymphomas of follicular histology. B1 stained the majority of cells in suspensions prepared from JK, SH, AT and SA, and together with the T cell monoclonal antibody accounted for more than 95% of the cells. A small population of cells expressing antigen determinants recognised by B1 and OKT11 was observed in SA. This population of cells is analogous to previously reported neoplastic B cell populations from a small number of malignant lymphomas, which express the T cell antigen recognised by OKT11, (Aisenberg *et al.*, 1981). FMC4 stained the majority of cells in suspension from JK, SH, AT and SA and together with the T cell monoclonal antibody accounted for more than 85% of the cells. Neoplastic cell populations from all five cases expressed one or more determinants associated with B cells, consistent with these tumours being of B cell origin.

The monoclonal antibody J5, which reacts with cALL cells and some B and T cell lymphomas including neoplastic and normal B cells of follicle centres (Ritz *et al.*, 1980, 1981; Habeshaw *et al.*, 1983; Stein *et al.*, 1982), stained 53–72% of cells in suspensions from JK, SH, AT and SA. This antibody does not appear to react with T cells in follicular lymphomas (Stein *et al.*, 1982) and this finding is a further demonstration of the similarity of the neoplasms described in this report to other cases of follicular lymphoma.

The majority of cells in suspension (82–97%) from JK, SH, AT and SA were negative for the B cell subset antibody FMC 7, which reacts preferentially with neoplastic cells in prolymphocytic leukaemia and variably with neoplastic cells in lymphoma (Catovsky *et al.*, 1981; Collins *et al.*, 1983). The monoclonal antibody RF-A1 which recognises T cells and a shared determinant present on a normal B cell subset and neoplastic B cells of CLL (Martin *et al.*, 1981) reacted with more cells than expected when tested with cell suspensions prepared from the four cases. A similar observation for cb/cc lymphoma of follicular histology has been reported by Habeshaw *et al.* (1983).

All of the lymphomas in this study showed histology typical of follicle centre cell lymphoma, cb/cc follicular. Lymphomas with this histology in which the neoplastic cells do not express sIg are rare and represent less than 3% of follicular tumours in our series. In a recent immunohistological study of NHL Tubbs *et al.* (1984) found one Ig negative lymphoma with a follicular histology in a group of 53 such cases examined, a similar incidence to that in our own series.

Human "null" lymphocytes defined by a lack of T lymphocyte markers and Ig expression have been identified among mononuclear cells from normal peripheral blood, lymph nodes and spleen. A brief review of the earlier literature relating to this subject can be found in Haegert & Coombs (1979). In this article and a subsequent paper (Haegert, 1981) it is claimed that the majority of "null" lymphocytes are of B cell lineage by virtue of surface Ig demonstrable by the mixed antiglobulin technique. Our earlier studies (Payne *et al.*, 1977) and the data relating to normal lymph node populations presented in this paper support this conclusion. Nevertheless whether all "null" cells detected in these tissues bear sIg and are therefore of B cell lineage remains to be resolved. Technical improvements such as those described by Haegert (1981) and Cordell *et al.* (1984) together with the application of T and B cell specific monoclonal antibodies will help to resolve this problem.

Neoplastic "null" cells occur in ALL, CLL and NHL. "Null" cells in CLL can be shown to be capable of synthesizing Ig *in vitro* (Gordon *et al.*, 1978) thus providing evidence for their B cell origin (Gordon, 1984). We have also shown that isolated neoplastic cells from cases of follicular NHL secrete whole Ig and/or free Ig light chains when cultured *in vitro* (Hannam-Harris *et al.*, 1982; Stevenson *et al.*, 1984). Neoplastic cells from four cases in this study were investigated for their ability to secrete Ig. Neoplastic cells from two of these, AT and SH secreted monotypic Ig light chain but did not secrete whole Ig *in vitro*. This pattern of Ig secretion is unusual in follicular NHL and has been associated with immature normal and neoplastic B lymphocyte populations in CLL (Hannam-Harris *et al.*, 1980; Gordon *et al.*, 1983).

In ALL the majority of "null" cells can be shown to be of B cell lineage by the presence of cytoplasmic $\mu$ chain (Vogler *et al.*, 1978) or by gene rearrangement (Korsmeyer *et al.*, 1981). Studies of Ig gene rearrangement have been helpful in determining the monoclonality of lymphoid tumours, including those of follicular histology, where this cannot be established by Ig phenotypic analysis (Arnold *et al.*, 1983). Similarly this technique has been used to establish the monoclonality of lymphomas in transplant patients (Cleary *et al.*, 1984). A JH probe has been used to demonstrate Ig gene rearrangement in the neoplastic cells isolated from one case included in this study (SA), strongly supporting a neoplastic B cell origin, (Dr Nigel O'Connor, personal communication).

We have shown by a variety of techniques that the neoplastic cells from the lymphomas described in this report are of B cell origin. However we are not able to decide on the basis of the present investigations, whether these cells are at an early stage of B cell maturation. These tumours may represent a separate developmental lineage of B lymphocytes within follicle centres or alternatively asynchrony between Ig expression and homing to sites of B cell development, analogous to that reported in myeloid neoplasms for the development of cytoplasmic enzymes and surface antigen expression (Scott *et al.*, 1982). Alternatively failure to express Ig by the majority of the neoplastic cells may arise as a consequence of an acquired defect in production by mechanisms similar to that observed in myeloma (Williams *et al.*, 1966), by aberrant Ig gene rearrangement (McIntosh *et al.*, 1983) or by factors unknown.

This report serves to demonstrate that a co-ordinated approach is required for the investigation of B cell neoplasms. If routine immunohistology and immunological analysis of cell suspensions fails to demonstrate the cell of origin, functional studies as described in this report may be useful. However, in a small number of cases where the B cell origin remains undetermined the ultimate investigation would appear to be Ig gene rearrangement.

We acknowledge the Leukaemia Research Fund for financial support.

### References

AISENBERG, A.C., BLOCK, K.J. & WILKES, B.M. (1981). Malignant lymphoma with dual B and T cell markers. *J. Exp. Med.*, **154**, 1709.

ARNOLD, A., COSSMAN, J., BAKHSHI, A., JAFFE, E.S., WALDMANN, T.A. & KORSMEYER, S.J. (1983). Immunoglobulin gene rearrangements as unique clonal markers in human lymphoid neoplasms. *N. Engl. J. Med.*, **309**, 1593.

BECKMAN, I.G.R., BRADLEY, J., BROOKS, D.A. & 4 others. (1980). Human lymphocytic markers defined by antibodies derived by somatic cell hybrids. II A hybridoma secreting antibody against an antigen expressed by human B cell and null lymphocytes. *Clin. Exp. Immunol.*, **40**, 593.

BEVERLEY, P.C.L. & CALLARD, R.E. (1981). Distinctive functional characteristics of human T lymphocytes defined by E Rosetting or a monoclonal anti-T cell antibody. *Eur. J. Immunol.*, **11**, 329.

BURROWS, P., LEJEUNE, M. & KEARNEY, J.F. (1979). Evidence that murine pre-B cells synthesise heavy chains but no light chains. *Nature*, **280**, 838.

CALIGARIS-CAPPIS, F., GOBBI, M., BOFILL, M. & JANOSSY, G. (1982). Infrequent normal B lymphocytes express features of B chronic lymphocytic leukaemia. *J. Exp. Med.*, **155**, 623.

CATOVSKY, D., CHERCHI, M., MATUTESE, E., BROOKS, D., BRADLEY, J. & ZOLA, H. (1981). Surface marker studies in B cell leukaemias with the monoclonal antibody FMC7. *Br. J. Haematol.*, **49**, 137.

CLEARY, M.L., WARNKE, R. & SKLAR, J. (1984). Monoclonality of lymphoproliferative lesions in cardiac-transplant recipients. Clonal analysis based on Immunoglobulin gene rearrangements. *N. Engl. J. Med.*, **310**, 477.

COOPER, A.G., BROWN, M.C., DERBY, H.A. & WORTIS, H.H. (1973). Quantitation of surface-membrane and intracellular gamma, mu and kappa chains of normal and neoplastic human lymphocytes. *Clin. Exp. Immunol.*, **13**, 481.

COLLINS, R.J., BUNCE, I.H., CLARKE, E.C. & 9 others. (1983). Malignant lymphoma reactive with the monoclonal antibody FMC 7. *Pathology*, **15**, 350.

CORDELL, J.L., FALINI, B., ERBER, W.N. & 6 others. (1984). Immunoenzymatic labelling of monoclonal antibodies using immune complexes of alkaline phosphatase and monoclonal anti-alkaline phosphatase (APAAP Complexes). *J. Histochem. Cytochem.*, **32**, 219.

ENGVALL, E. & PERLMAN, P. (1972). Enzyme linked immunosorbent assay, Elisa III. Quantitation of specific antibodies by enzyme labelled anti-immunoglobulin in antigen coated tubes. *J. Immunol.*, **109**, 129.

GERARD-MARCHANT, R., HAMLIN, L., LENNERT, K., RILKE, F., STANSFELD, A.S. & VAN UNNIK, J.A.M. (1974). Classification of non-Hodgkin's lymphomas. *Lancet*, **ii**, 406.

GODAL, T. & FUNDERUND, S. (1982). Human B-cell neoplasms in relation to normal B-cell differentiation and maturation processes. *Adv. Cancer Res.*, **36**, 211.

GORDON, J. (1984). Molecular aspects of immunoglobulin expression by human B cell leukaemias and lymphomas. *Adv. Cancer Res.*, **41**, 71.

GORDON, J., HOWLETT, A.R. & SMITH, J.L. (1978). Free light chain synthesis by neoplastic cells in chronic lymphocytic leukaemia and non-Hodgkin's lymphoma. *Immunology*, **34**, 397.

GORDON, J., MELLSTEDT, H., AMAN, P., BIBERFIELD, P., BJORKHOLM, M. & KLEIN, G. (1983). Phenotypes in chronic B lymphocytic leukaemia probed by monoclonal antibodies and immunoglobulin secretion studies: Identification of stages of maturation arrest and the relation to clinical findings. *Blood*, **62**, 910.

GROBLER, P., BUERKI, H., COTTIER, H., HESS, M.W. & STONER, R.D. (1974). Cellular bases for relative radioresistance of the antibody-forming system at advanced stages of the secondary response to tetanus toxoid in mice. *J. Immunol.*, **112**, 2154.

HABESHAW, J.A., BAILEY, D., STANSFELD, A.G. & GREAVES, M.F. (1983). The cellular content of non Hodgkin lymphomas: A comprehensive analysis using monoclonal antibodies and other surface marker techniques. *Br. J. Cancer*, **47**, 327.

HAEGERT, D.G. (1981). The mixed antiglobulin rosetting reaction (MARR) and direct antiglobulin rosetting reaction (DARR): sensitive tests for demonstration of immunoglobulin-bearing B lymphocytes. *J. Immunol.*, **41**, 1.

HAEGERT, D.G. & COOMBS, R.R.A. (1979). Do human B and null lymphocytes form a single immunoglobulin bearing population. *Lancet*, **ii**, 1051.

HANNAM-HARRIS, A.C., GORDON, J. & SMITH, J.L. (1980). Immunoglobulin synthesis by neoplastic B lymphocytes: Free light chain synthesis as a marker of B cell differentiation. *J. Immunol.*, **125**, 2177.

HANNAM-HARRIS, A.C., GORDON, J., WRIGHT, D.H. & SMITH, J.L. (1982). Correlation between Ig-synthesis patterns and lymphoma classification. *Br. J. Cancer*, **46**, 167.

HOGG, N., ROSS, G.D., JONES, D.B., SLUSARENKO, M., WALPORT, M.J. & LACHMAN, P.J. (1984). Identification of an anti-monocyte monoclonal antibody that is specific for membrane complement receptor type one ($CR_1$). *Eur. J. Immunol.*, **14**, 236.

HSU, S., CROSSMAN, J. & JAFFE, E. (1983). Lymphocyte subsets in normal human lymphoid tissue. *Am. J. Clin. Pathol.*, **80**, 21.

ISAACSON, P., WRIGHT, D.H., JUDD, M.A., JONES, D.B. & PAYNE, S.V. (1980). The nature of the immunoglobulin containing cells in malignant lymphoma: An immunoperoxidase study. *J. Histochem. Cytochem.*, **28**, 761.

KORSMEYER, S.J., HEITER, P.A., RAVETCH, J.V., POPLACK, D.G., WALDMAN, T.A. & LEDER, P. (1981). Developmental hierachy of immunoglobulin gene rearrangements in human leukaemic pre B cells. *Proc. Natl Acad. Sci.*, **78**, 7096.

LEECH, J.H., CLICK, A.D., WALDRON, J.A., FLEXNER, J.M., HORN, R.P. & COLLINS, R.D. (1975). Malignant lymphomas of follicular centre cell origin in man. I Immunologic Studies. *J. Natl Cancer Inst.*, **54**, 11.

McINTOSH, R.V., COHEN, B.B., STEEL, C.M., READ, H., MOXLEY, M. & EVANS, H.J. (1983). Evidence for involvement of the immunoglobulin heavy chain locus in 8:14 translocation of human B lymphomas. *Br. J. Cancer*, **31**, 475.

MARTIN, P.J., HANSEN, J.A., SIADAK, A.W. & NOWINSKY, R.C. (1981). Monoclonal antibodies recognizing human T lymphocytes and malignant human B lymphocytes: A comparative study. *J. Immunol.*, **127**, 1920.

MELCHERS, F., ANDERSON, J., CORBAL, C. & 4 others. (1982). Regulation of B lymphocyte replication and maturation. *J. Cell Biochem.*, **19**, 315.

MEPHEM, B.L., FRATER, W. & MITCHELL, B.S. (1979). The use of proteolytic enzymes to compare immunoglobulin staining by the PAP technique. *Histochem. J.*, **11**, 345.

MORRISON, M., BAYSE, G.S. & WEBSTER, R.G. (1971). Use of lactoperoxidase catalysed iodination in immunochemical studies. *Immunochemistry*, **8**, 289.

OSMOND, D.G. & NOSSAL, G.J.V. (1974). Differentiation of lymphocytes in mouse bone marrow. II Kinetics of maturation and renewal of antiglobulin binding cells studies by double labelling. *Cell Immunol.*, **13**, 132.

PAYNE, S.V., SMITH, J.L., JONES, D.B. & WRIGHT, D.H. (1977). Lymphocyte markers in non-Hodgkin's lymphomas. *Br. J. Cancer*, **36**, 57.

PRIEUR, A.M. & BROUET, J.C. (1974). Membrane markers on chronic lymphocytic leukaemia cells: A B cell leukaemia with rosettes to anti-sheep erythrocytes. Antibody activity of the membrane bound IgM and T cell leukaemia with surface Ig. *Clin. Immunol. Immunopathol.*, **2**, 481.

RAFF, M.C., MEGSON, M., OWEN, J.J.T. & COOPER, M.D. (1976). Early production of IgM by B lymphocyte precursors in mouse. *Nature*, **259**, 224.

RITZ, J., NADLER, L.M., BHAN, A.K., NOTIS-McCONARTY, J., PESANDO, J.M. & SCHLOSSMAN, S.F. (1981). Expression of common acute lymphoblastic leukaemia antigen (cALL) by lymphomas of B and T cell lineage. *Blood*, **58**, 648.

RITZ, J., PESANDO, J.M., NOTIS-McCONARTY, J., LAZARIS, H. & SCHLOSSMAN, S.F. (1980). A monoclonal antibody to human acute lymphoblastic leukaemia antigen. *Nature*, **283**. 583.

SCOTT, C.S., BYNDE, A.G., ROBERTS, B.E. & HOUGH, D. (1982). Asynchronous expression of granulocyte membrane receptors in myeloid neoplasms. *Br. J. Haematol.*, **52**, 439.

STASHENKO, P., NADLER, L.M., HARDY, R. & SCHLOSSMAN, S.F. (1980). Characterization of a human B lymphocyte-specific antigen. *J. Immunol.*, **125**, 1678.

STEIN, H., BONK, A., TOLKSDORF, G., LENNERT, K., RODT, H. & GERDES, J. (1980). Immunohistologic analysis of the organization of normal lymphoid tissue and non-Hodgkin's lymphomas. *J. of Histochemistry and Cytochemistry*, **28**, 746.

STEIN, H., GERDES, J. & MASON, D.Y. (1982). The normal and malignant germinal centre. *Clin. Haematol.*, **11**, 531.

STEVENSON, F.K., HAMBLIN, T.J. & STEVENSON, G.T. (1981). The nature of immunoglobulin G on the surface of B lymphocytes in chronic lymphocytic leukaemia. *J. Exp. Med.*, **154**, 1965.

STEVENSON, F.K.,HAMBLIN, T.J., STEVENSON, G.T. & TUTT, A.L. (1980). Extracellular idiotypic immunoglobulin arising from human leukaemic B lymphocytes. *J. Exp. Med.*, **152**, 1484.

STEVENSON, F.K., GREGG, E.O., SMITH, J.L. & STEVENSON, G.T. (1984). Secretion of immunoglobulin by neoplastic B lymphocytes from lymph nodes of patients with lymphoma. *Br. J. Cancer*, **50**.

STEVENSON, G.T., SMITH, J.L. & HAMBLIN, T.J. (1983). *Immunological Investigation of Lymphoid Neoplasms.* Edinburgh/London: Churchill Livingstone.

TUBBS, R.R., FISHLEDER, A., WEISS, R.A., SAVAGE, R.A., SEBEK, B.A. & WEICK, J.K. (1983). Immunohistologic cellular phenotypes of lymphoproliferative disorders. *Am. J. Pathol.,* **113,** 207.

VERBI, W., GREAVES, M.F., SCHNEIDER, C. & 5 others. (1982). Monoclonal antibodies OKT 11 and OKT 11A have pan-T reactivity and block sheep erythrocyte "receptors". *Eur. J. Immunol.,* **12,** 81.

VOGLER, L.B., CRIST, W.M., BOCKMAN, D.E., PEARL, E.R., LAWTON, A.R. & COOPER, M.D. (1978). Pre B cell leukaemia: A new phenotype of childhood lymphoblastic leukaemia. *N. Engl. J. Med.,* **298,** 872.

WAKEFIELD, J.D. & THORBECKE, G.J. (1968). Relationship of germinal centres in lymphoid tissue to immunological memory. The detection of primed cells and their proliferation upon cell transfer to lethally irradiated syngeneic mice. *J. Exp. Med.,* **128,** 171.

WILLIAMS, R.C., BRUNING, R.D. & WOLLHEIM, F.A. (1966). Light chain disease: An abortive variant of multiple myeloma. *Ann. Intern. Med.,* **65,** 471.